# Supplementary material for: Content-rich biological network constructed by mining PubMed abstracts
Source: BMC Bioinformatics. 2004 Oct 8;5:147. doi: 10.1186/1471-2105-5-147 (PMC528731; doi:10.1186/1471-2105-5-147)
Supplement: Additional File 2 — The original results of the above study (non-essential files are deleted to keep the file size under the limit set by BMC bioinformatics). [file 1471-2105-5-147-S2.bz2 › chilibotAdditionalFile2/dip20/375ID3037029E9781/html/NGFR_NGFB.html]

 


 **NGFR** and **NGFB** 
  
Found 1676 abstracts in PubMed, retrieved 20.  
 

 What does Google say? 
 PDF only 
| .edu only 

---

**Interactive relationship** (e.g. stimulation, inhibition, etc)

**Inhibitory relationship**- Pathways responsible for transducing the sensitisation of TRPV1 by TrkA, the NGF  [ **NGFB** ]  receptor  [ **NGFR** ] , were characterised by observing the effects of inhibitors of key members of NGF  [ **NGFB** ]  activated second messenger signalling cascades.  Ref: 12815188 J Physiol< MedlineTA>J Physiol,
**Neutral relationship**- NADE p75NTR  [ **NGFR** ]  associated cell death executor is a p75NTR  [ **NGFR** ]  associated protein that mediates apoptosis in response to NGF  [ **NGFB** ]  by interacting with the cell death domain of p75NTR  [ **NGFR** ] .  Ref: 12852261 Vitam Horm, 2003
- Here we show that survival of sympathetic neurons supported by NGF  [ **NGFB** ]  on distal axons requires the kinase activity of the NGF  [ **NGFB** ]  receptor  [ **NGFR** ] , TrkA, in both distal axons and cell bodies.  Ref: 12848932 Neuron, 2003
- The p75 neurotrophin receptor p75NTR  [ **NGFR** ]  is a member of the tumor necrosis factor receptor TNFR superfamily, and can mediate both cell survival and cell death in response to nerve growth factor  [ **NGFB** ]  NGF  [ **NGFB** ] .  Ref: 12852261 Vitam Horm, 2003

**Non-interactive relationship** (e.g. studied together, co-existance, homology, etc.)

- Human nerve growth factor  [ **NGFB** ]  receptor  [ **NGFR** ]  and cytosine deaminase fusion genes.  Ref: 12869218 Hum Gene Ther, 2003
- Expression of low affinity nerve growth factor  [ **NGFB** ]  receptor  [ **NGFR** ]  p75 and its regulation in spinal motor neurons of aged rats following axonal injury were investigated by immunocytochemical staining with antibody against p75.  Ref: 12876432 Dev Neurosci, 2003
- We present the results of cosegregation analysis for the following loci the gene for the Na, K ATPase alpha 1 subunit isoform Atp1a1, the endothelin 2 gene Edn2, the low affinity nerve growth factor  [ **NGFB** ]  receptor  [ **NGFR** ]  gene Lngfr, and a region of chromosome 10 marked with the D10Rat58 microsatellile located 3 cM away of the aldolase C gene AldC.  Ref: 12884521 Genetika, 2003
- In both NGF  [ **NGFB** ]  transgenic and nontransgenic mice with hypomorphic expression of p75NTR  [ **NGFR** ] , there is a significant loss of DRG neurons compared to mice with normal p75NTR  [ **NGFR** ]  expression.  Ref: 12887413 Eur J Neurosci, 2003
- This distribution is related to the spatial distribution of nerve endings identified by labeling of the low affinity nerve growth factor  [ **NGFB** ]  NGF  [ **NGFB** ]  receptor  [ **NGFR** ]  p75 NTR.  Ref: 12821716 J Dent Res, 2003
- We could show that the viral latent membrane protein 1 LMP1 is responsible for the induction of CD83 by using an LCL expressing a ligand or antibody inducible recombinant nerve growth factor  [ **NGFB** ]  receptor  [ **NGFR** ]  LMP1 fusion protein.  Ref: 12857898 J VirolJ Virol, 2003
- CONCLUSIONS The results indicate that coexpression of both low and high affinity NGF  [ **NGFB** ]  receptors are not only more efficient in restoration of NGF  [ **NGFB** ]  induced differentiation pathway, but also be able to activate the pro apoptotic activity of low affinity NGF  [ **NGFB** ]  receptor  [ **NGFR** ]  and make the tumor cells become NGF  [ **NGFB** ]  dependent and irreversibly differentiated.  Ref: 12877805 Chin Med J (Engl), 2003
- Comparisons of calcitonin gene related peptide immunoreactivity in the dorsal horn revealed that the area occupied by DRG central processes was not significantly different between p75NTR  [ **NGFR** ]  hypomorphic mice and wild type siblings, or between NGF  [ **NGFB** ]  transgenic mice with either hypomorphic or normal expression of p75NTR  [ **NGFR** ] .  Ref: 12887413 Eur J Neurosci, 2003
- An 11 mer antisense PNA directed at the initiation codon dose dependently inhibited p75NTR  [ **NGFR** ]  expression and death signalling by nerve growth factor  [ **NGFB** ]  in Schwann cell cultures.  Ref: 12824038 Bioorg Med Chem Lett, 2003
- We propose that DRG central processes arborize extensively in both NGF  [ **NGFB** ]  transgenic and nontransgenic p75NTR  [ **NGFR** ]  hypomorphic mice in order to compensate for the loss of DRG neurons and restore dorsal horn innervation to normal levels.  Ref: 12887413 Eur J Neurosci, 2003
- A murine model of X linked chronic granulomatous disease X CGD, an inherited immune deficiency with absent phagocyte NADPH oxidase activity caused by defects in the gp91 phox gene, was used to evaluate a bicistronic retroviral vector in which expression of human gp91 phox and a linked gene for DeltaLNGFR, a truncated form of human low affinity nerve growth factor  [ **NGFB** ]  receptor  [ **NGFR** ] , are under the control of a spleen focus forming virus long terminal repeat LTR.  Ref: 12804147 Hum Gene Ther, 2003
- In addition, tumors were examined for expression of vimentin, cytokeratin, smooth muscle actin, desmin, melan A, neural cell adhesion molecule, S 100, glial fibrillary acidic protein, nerve growth factor  [ **NGFB** ]  receptor  [ **NGFR** ] , and collagen type IV.  Ref: 12824506 Vet Pathol, 2003
- The 75 kDa neurotrophin receptor p75NTR  [ **NGFR** ]  and two neurotrophin receptor homologs NRH1, NRH2 constitute a subfamily of the nerve growth factor  [ **NGFB** ]  tumor necrosis factor receptor superfamily.  Ref: 12843241 J Neurosci, 2003
- However, NGF  [ **NGFB** ]  rescue from growth factor withdrawal requires NGF  [ **NGFB** ]  signalling through the high affinity tyrosine kinase receptor TrkA, while NGF  [ **NGFB** ]  rescue from TNFalpha promoted cell death requires NGF  [ **NGFB** ]  signalling through the low affinity p75NTR  [ **NGFR** ]  receptor.  Ref: 12882321 Aging Cell, 2003
- Quantitative in situ hybridization was used to detect changes in neuronal PACAP mRNA expression and to correlate this expression with neuronal mRNA expression of the NGF  [ **NGFB** ]  receptor  [ **NGFR** ]  tyrosine kinase trk A.  Ref: 12890505 Neuroscience, 2003
- This study examined the roles of nerve growth factor  [ **NGFB** ]  NGF  [ **NGFB** ]  and the p75 neurotrophin receptor p75NTR  [ **NGFR** ]  in the growth of dorsal root ganglion DRG central processes in the dorsal horn.  Ref: 12887413 Eur J Neurosci, 2003
- In this review, we focus on the proapoptotic actions of NADE that regulate p75NTR  [ **NGFR** ]  signaling in response to NGF  [ **NGFB** ] .  Ref: 12852261 Vitam Horm, 2003
- Expression and Role of Low Affinity Nerve Growth Factor  [ **NGFB** ]  Receptor  [ **NGFR** ]  p75 in Spinal Motor Neurons of Aged Rats following Axonal Injury.  Ref: 12876432 Dev Neurosci, 2003
- Two genetically modified mouse strains were used transgenic mice that overexpress NGF  [ **NGFB** ]  in the CNS under the control of the glial fibrillary acidic protein promoter, and p75NTR  [ **NGFR** ]  exon III null mutant mice that express a hypomorphic form of this receptor.  Ref: 12887413 Eur J Neurosci, 2003
- In this paper, genetic constructs were designed to express the CD enzyme fused to the transmembrane and extracellular domains of the human nerve growth factor  [ **NGFB** ]  receptor  [ **NGFR** ]   **NGFR** , thus allowing for positive identification of transduced cells by flow cytometry and positive selection by magnetic bead technology.  Ref: 12869218 Hum Gene Ther, 2003
- Although we demonstrate the expressions of several markers, such as CD10, CD56, CD99, factor XIII, and low affinity nerve growth factor  [ **NGFB** ]  receptor  [ **NGFR** ] , in the spindle cells of the whorls for the first time, the lineage of the whorls still cannot be addressed due to the fact that these markers are lineage nonspecific.  Ref: 12833576 Yonsei Med J, 2003
- the lack of NGF  [ **NGFB** ]  receptor  [ **NGFR** ]  expression on some astrocytes suggests heterogeneity of astroglia population.  Ref: 12849738 Neuroscience, 2003
- High affinity NGF  [ **NGFB** ]  receptor  [ **NGFR** ]  in the rat spinal cord during acute and chronic phases of experimental autoimmune encephalomyelitis a possible functional significance.  Ref: 12825322 Arch Ital Biol, 2003
- Immunohistochemical staining of the explanted cords revealed that the surviving cells stained positively for nerve growth factor  [ **NGFB** ]  receptor  [ **NGFR** ] .  Ref: 12820682 J Neurotrauma, 2003
- METHODS Retroviral vectors were constructed to express the high affinity NGF  [ **NGFB** ]  receptor  [ **NGFR** ]  TrkA and low affinity NGF  [ **NGFB** ]  receptor  [ **NGFR** ]  p75 NTR.  Ref: 12877805 Chin Med J (Engl), 2003
- The cells comprising whorls showed positive reactions for CD10, CD56, CD99, factor XIII, and low affinity nerve growth factor  [ **NGFB** ]  receptor  [ **NGFR** ]  in addition to vimentin and alpha smooth muscle actin.  Ref: 12833576 Yonsei Med J, 2003
